# Adjunctive embolization of target vessel dissections to improve suitability for fenestrated-branched endovascular aortic repair

**DOI:** 10.1016/j.jvscit.2026.102366

**Published:** 2026-06-20

**Authors:** Zemia C. Ferreira, Hannah V. Oden-Brunson, Ying Huang, Steven Maximus, Naveed Saqib, Erin Greenleaf, Thanila A. Macedo, Gustavo S. Oderich

**Affiliations:** a Advanced Endovascular Aortic Research Program and the Marcus VITAL Laboratory, Division of Vascular Surgery and Endovascular Therapy, Michael E. DeBakey Department of Surgery, Baylor College of Medicine, Houston, TX; b Department of Cardiothoracic and Vascular Surgery, The University of Texas Health Science Center at Houston, Houston, TX

**Keywords:** Postdissection thoracoabdominal aortic aneurysm, False lumen embolization, Fenestrated-branched endovascular aortic repair, Target vessel dissection, Superior mesenteric artery

## Abstract

Suitability of renal-mesenteric target arteries is an essential criterion for fenestrated-branched endovascular aortic repair (FB-EVAR). We describe a staged adjunctive technique of target vessel false lumen (FL) embolization to improve suitability for FB-EVAR in patients with chronic postdissection thoracoabdominal aortic aneurysms (PD-TAAA) and dissection extension into the superior mesenteric artery (SMA). Three patients with SMA dissection and ectasia (diameter > 12-14 mm) underwent staged FL embolization with pre-emptive stent placement to optimize SMA incorporation. Using bilateral transfemoral access with intravascular ultrasound guidance, the proximal SMA true lumen was stented with an 8 L × 39 mm VBX balloon-expandable stent graft (W. L. Gore & Associates) coupled with a 14 × 60 mm bare-metal self-expandable stent extending past the dissection flap reentrance. The SMA FL was then embolized using 15 mm IMPEDE-FX polymer plugs (Shape Memory Medical), followed by postdilatation with a 12 to 14 mm angioplasty balloon. FB-EVAR was completed as a second-stage procedure without complications. Follow-up computed tomography angiography confirmed successful target vessel incorporation without endoleak in all three patients. Staged adjunctive target vessel FL embolization is technically feasible and improves suitability for FB-EVAR in patients with postdissection thoracoabdominal aortic aneurysms and dissected SMA. Larger series and longer follow-up are needed to assess durability and secondary complications.

Fenestrated-branched endovascular aortic repair (FB-EVAR) has been increasingly used in patients with chronic postdissection thoracoabdominal aortic aneurysms (PD-TAAA). Several single- and multicenter studies have shown high-technical success and low morbidity and mortality, comparable to degenerative aneurysms and, in several studies, lower than historical results of open surgical repair.^1^, ^2^, ^3^ In addition to the presence of suitable proximal and distal sealing zones in the aorta and/or iliac arteries, endovascular incorporation of renal and mesenteric target vessels requires the presence of a nondissected arterial segment with adequate diameter and length to accommodate a bridging stent graft.^4^, ^5^, ^6^ It is estimated that 30% to 50% of patients have extension of the dissection into at least one of the renal and mesenteric arteries.^1^^,^^3^^,^^7^, ^8^, ^9^ In most patients, standard target vessel incorporation can be achieved by extending the bridging stent beyond the distal re-entry tear. However, in patients with marked target vessel ectasia (>12-14 mm), circumferential apposition of the bridging stent graft may be compromised, resulting in persistent false lumen (FL) perfusion and risk of type IC endoleak. In these patients, presence of an ectatic and dissected target vessel may render the anatomy unsuitable for endovascular repair.

We describe a technique of staged adjunctive target vessel FL embolization to improve suitability of mesenteric artery incorporation during FB-EVAR of PD-TAAA in patients with distal extension of the dissection flap into the superior mesenteric artery (SMA) and vessel ectasia. This article summarizes the technical pitfalls and preliminary experience in three patients.

## Methods

The study was approved by the Institutional Review Board of the two participating institutions. All patients consented for enrollment on a prospective, nonrandomized sponsor-initiated protocol to evaluate FB-EVAR of complex aortic aneurysms (NCT01937949).

Patient demographics, cardiovascular risk factors, anatomical details [such as true lumen (TL) diameters at relevant levels, branch origins from TL vs FL, and extent of dissection into visceral branches], procedural characteristics (such as device sizes, fluoroscopy time, and contrast volume), and early outcomes were collected prospectively and recorded in case report forms. Variables and clinical outcomes were reported in accordance with the proposed reporting standards of the Society for Vascular Surgery for endovascular repair of aneurysms involving the renal and mesenteric arteries.^4^

### Patients

**Fig 1:**
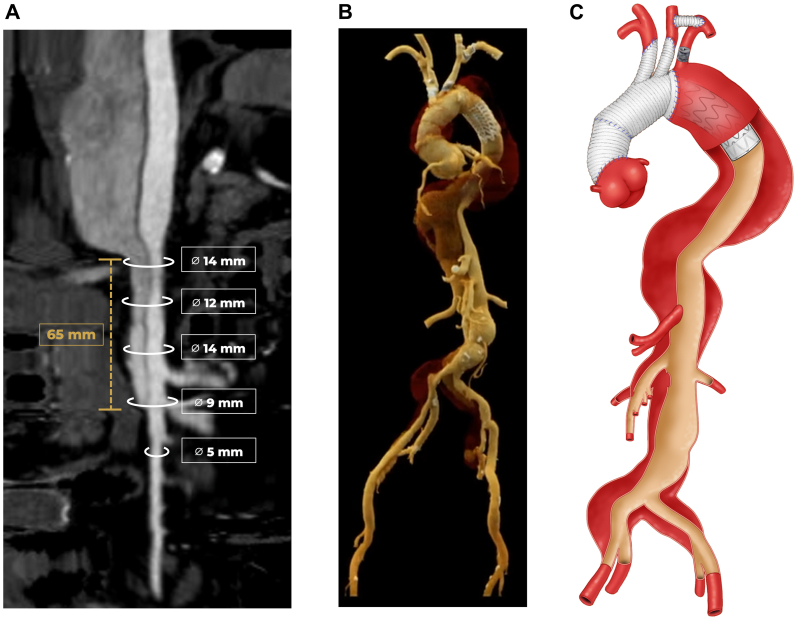
Preoperative imaging of a patient with postdissection thoracoabdominal aneurysm (PD-TAAA). **A,** Centerline reconstruction of the superior mesenteric artery (SMA) demonstrating extension of the dissection into the vessel and diameters along the vessel. **B,** Three-dimensional reconstruction illustrates the extent II PD-TAAA and overall aortic anatomy. **C,** Schematic illustration of the patient-specific anatomy, demonstrating extension of the dissection into the SMA.

A total of 640 patients underwent FB-EVAR in the sponsor-investigator investigational device exemption study. Of these, 97 patients (15.3%) were treated for chronic PD-TAAAs. Three patients (0.47%) underwent staged adjunctive FL embolization at the SMA to improve suitability and sealing at the target vessel. Two patients had the adjunctive procedure performed before FB-EVAR due to unsuitable seal zone at the SMA (Fig 1), and one patient underwent a secondary intervention after FB-EVAR to treat a Type IC endoleak originating from the SMA branch FL. All three patients had extent II TAAAs with a median diameter of 67 mm (range, 59-74 mm). The SMA diameter at the sealing zone averaged 14 mm (range, 9-17 mm) with the dissection flap extending >5 cm distal from the aortic origin (Table I).

**Table I:** Demographics, cardiovascular risk factors, and anatomical features

Variables	No (%) or median (range)
Demographics	
Age, years	72 (63-77)
Male sex	3 (100)
Cardiovascular risk factors	
Hyperlipidemia	3 (100)
Smoking history	0
Coronary artery disease	2 (66.7)
COPD	0
Peripheral artery disease	0
Dialysis	0
Diabetes	0
Chronic heart failure	0
Hypertension	3 (100)
Risk assessment	
ASA class ≥ 3	2 (66.7)
Aortic history	
Previous aortic repair	
Open repair	3 (100)
Endovascular repair	2 (66.7)
Family history of aortic disease	0
GTAD	0
Anatomical characteristics	
Extent of aneurysm	
CAAA	0
TAAA II	3 (100)
Largest aortic diameter, mm	67 (59-74)
Chronic aortic dissection	3 (100)
Status of aneurysm	
Symptomatic Aneurysm^a^	0
Elective intact aneurysm	3 (100)
Status of superior mesenteric artery	
Patent	3 (100)
Stenosis > 50%	0
Diameter, mm	14 (9-17)

*ASA*, American Society of Anesthesiologists; *CAAA*, complex abdominal aortic aneurysm; *CKD*, chronic kidney disease; *COPD*, chronic obstructive pulmonary disease; *EVAR*, endovascular aortic repair; *GTAD*, genetically triggered aortic disease; *IQR*, interquartile range; *TAAA*, thoracoabdominal aortic aneurysm; *TIA*, transient ischemic attack. Categorical data are shown as number (%).

a Patients presenting abdominal pain, contained aneurysm rupture, or aneurysm rupture with hypotension.

### Technique

**Fig 2:**
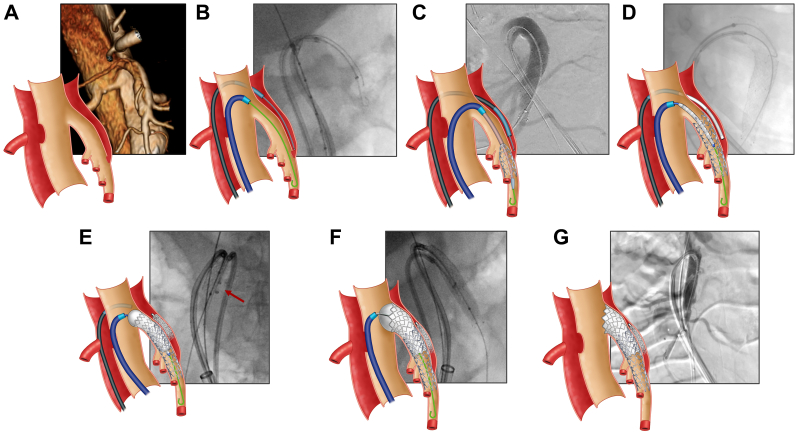
Procedural steps of the adjunctive false lumen (FL) embolization technique. **A,** Preoperative three-dimensional volume-rendered computed tomography angiography (CTA) demonstrating dissection involving the superior mesenteric artery (SMA). **B,** Dual femoral access is obtained, followed by selective catheterization of the true lumen (TL) and FL under fluoroscopic and intravascular ultrasound (IVUS) guidance. **C,** Through the TL, a distal self-expanding bare-metal stent is deployed to scaffold the dissected segment. **D,** A proximal balloon-expandable covered stent is then positioned and deployed within the TL to achieve sealed relining from the branch origin to the proximal main trunk, whereas a Shape Memory Polymer (SMP) plug is positioned within the FL. **E,** Via the FL sheath, the SMP plug is deployed to occlude the branch-level FL channel (*red arrow*). **F,** Postdilation of the covered stent is performed using a compliant balloon to achieve flaring at the branch origin and optimize apposition. **G,** Final digital subtraction angiography demonstrating widely patent SMA stent with preserved flow and no residual filling of the FL.

The procedure was performed in a hybrid operating room equipped with fixed imaging. The patient was positioned supine, and the abdomen and both groins were prepped and draped in a sterile fashion. Using duplex ultrasound guidance, bilateral retrograde transfemoral access was obtained and the patient was systemically heparinized. Access was established in both the TL and FL of the aorta. The TL of the dissected SMA was selectively catheterized using a 7F Oscor Destino steerable sheath (Integer Oscor Inc) advanced from the ipsilateral access. A Kumpe catheter (Cook Medical Inc) and a 0.035″ Glidewire (Terumo, Ashitaka Factory) were advanced via the SMA TL and exchanged for a 0.035″ Rosen wire (Cook Medical Inc). The FL of the dissected SMA was catheterized in a similar fashion from the contralateral transfemoral access, and a 6F High Flex Ansel sheath (Cook Medical Inc) was positioned alongside the steerable sheath (Fig 2). Adequate guidewire positioning in the TL and FL of the SMA was confirmed using a 0.014″ intravascular ultrasound advanced through the TL guidewire.

The SMA TL was stented using a 12 to 14 × 60 mm self-expanding bare-metal stent (eg, Zilver Vena; Cook Medical Inc), which was extended across the dissected segment and jejunal branches into a nondissected distal segment of the SMA, beyond the target vessel reentrance. The stent was used for scaffolding the dissected membrane. The proximal SMA was stented using an 8 L × 39 mm balloon-expandable covered stent graft (eg, VBX; W. L. Gore & Associates) with at least 1.5 cm overlap with the distal self-expanding bare metal stent. The covered stent graft was extended proximally to the level of the SMA aortic origin. The VBX stent graft was postdilated using a 12 to 14 mm × 2 cm balloon to match the nominal diameter of the proximal SMA. Following stenting of the SMA TL, the FL was embolized using 15-mm IMPEDE-FX polymer plugs (Shape Memory Medical), which were deployed in the FL alongside the bare metal and covered stent graft. A total of one to three polymer plugs were deployed. Selective completion SMA angiography confirmed adequate flow and opacification of the SMA and its branches with no evidence of nontarget embolization.

### Results

**Fig 3:**
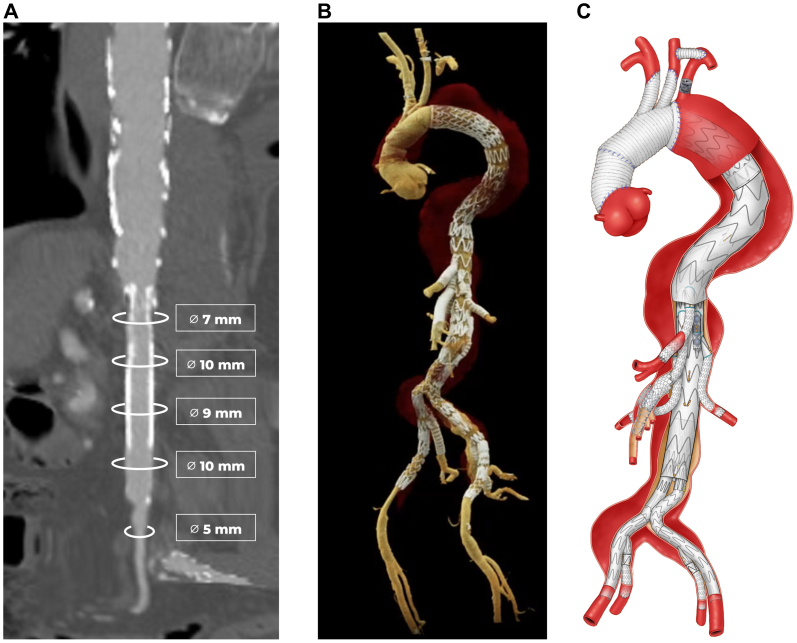
Three-month follow-up demonstrating successful sealing of the superior mesenteric artery (SMA) bridging stent graft with no evidence of type IC endoleak after fenestrated-branched endovascular aortic repair (FB-EVAR). **A,** Computed tomography angiography (CTA) demonstrating a patent SMA stent graft with adequate sealing and no evidence of type IC endoleak. **B,** Three-dimensional reconstruction illustrating the final post-FB-EVAR configuration. **C,** Schematic illustration depicting the final repaired anatomy.

Adjunctive SMA FL embolization and stenting were used in three patients with dissected SMA and chronic PD-TAAAs treated by FB-EVAR in the sponsor-investigator investigational device exemption study. Two patients had SMA TL stenting and FL embolization as an adjunctive procedure performed before FB-EVAR. In both patients, technical success was achieved with no evidence of SMA nontarget embolization and no postoperative complications. Follow-up at 3-months revealed no evidence of SMA Type IC endoleak and successful sealing of the SMA bridging stent graft (Fig 3). A third patient presented with persistent SMA type IC endoleak and aneurysm sac enlargement following the FB-EVAR. Dynamic computed tomography angiography confirmed that the endoleak originated from the distal SMA bridging stent graft seal zone, which was enlarged and affected by dissection, resulting in retrograde flow via reentrance to the aneurysm sac. A secondary intervention was performed under general endotracheal anesthesia with the patient in the posterolateral decubitus position. Using translumbar access, the aortic FL of the aorta and SMA FL were catheterized and excluded with multiple detachable coils. Intraoperative completion angiography and contrast-enhanced duplex ultrasound was obtained, confirming resolution of the type IC endoleak. Follow-up at 3 years confirmed no evidence of residual type I and III endoleak, but a persistent type II endoleak from lumbosacral vessels. The patient had no additional reinterventions (Table II).

**Table II:** Procedural details and clinical outcomes

Variables	Patient 1	Patient 2	Patient 3
Balloon expandable stent graft	VBX^a^ 8L × 39	VBX 11 × 39 and 11 × 59	VBX 11 × 59
Self-expandable stent graft	12 × 60	14 × 60 and 16 × 60	–
Embolic material (SMA false lumen)	Shape-form plug (IMPEDE FX^b^)	Shape-form plug (IMPEDE FX)	Detachable coils
Technical success	Yes	Yes	Yes
Early major adverse event (≤30 days)	No	No	No
30-day mortality	No	No	No
Late type I or III endoleak	No	No	No
SMA reintervention during follow-up	No	No	No

*SMA*, Superior mesenteric artery.

^a^VBX balloon expandable stent graft (W. L. Gore & Associates).

^b^IMPEDE FX polymer plug (Shape Memory Medical).

## Discussion

This article summarizes the technical pitfalls of adjunctive SMA FL embolization with TL stenting to optimize the target vessel sealing zone in patients considered unsuitable for FB-EVAR due to target vessel involvement by the dissection flap. It is estimated that 30% to 50% of patients with chronic postdissection aneurysms have involvement of at least one of the renal and mesenteric arteries by the dissection flap.^1^^,^^3^^,^^7^, ^8^, ^9^ Although in most patients dissection of a target vessel does not represent an absolute contraindication to endovascular incorporation and can be overcome by extending the covered stent graft beyond the re-entry tear, coverage of multiple side branches should be avoided. In patients with marked target vessel ectasia (>12-14 mm), however, standard bridging stent grafts may fail to achieve circumferential apposition, leaving a residual FL channel and resulting in type IC endoleak. The self-expanding bare metal stent was extended beyond the distal re-entry tear to prevent migration of the polymer plugs through the re-entry site into the SMA TL or its branches. The stent also provides scaffolding of the TL, and together with embolization of the SMA FL and postdilation of the TL covered stent graft, provides sealing to prevent a type IC endoleak from the target vessel via residual FL flow. We acknowledge that excessive distal extension may theoretically compromise FL-dependent jejunal branches, and that the extent of distal stenting should therefore be individualized according to branch anatomy and location of distal re-entry tears. Although no clinical or imaging evidence of bowel ischemia or branch loss was observed in our series, larger studies are necessary to better characterize this risk.

Other alternative methods to deal with a large and dissected SMA are a hybrid approach with replacement of a segment of the SMA with prosthetic graft to provide a sealing zone, or an extra-anatomic bypass. Although both these alternatives should be considered as part of the armamentarium, a total endovascular approach is appealing and may reduce the morbidity associated with open surgical reconstruction, particularly in the elderly or higher risk patients. Target vessel stenting and embolization may be performed in a single stage concomitantly with FB-EVAR; however, our preference is to perform the embolization as a staged intervention prior to FB-EVAR. In the event the endovascular technique is not successful, a hybrid approach or bypass may still be performed. Importantly, the durability of adjunctive FL embolization to provide long-lasting seal still needs to be determined. It is possible that further enlargement of the target vessel or lack of remodeling may lead to delayed endoleaks requiring secondary interventions.

Although our preliminary experience has been limited to the SMA, this technique can be applied to any target vessel if anatomically indicated. In our experience, the SMA more frequently demonstrated extensive dissection with marked ectasia, creating challenges for distal seal during FB-EVAR that were not as frequently encountered in renal or other target vessels.

The evolving role of FL embolization in patients with chronic dissections has been recently emphasized, both to prevent recalcitrant endoleaks as well as to optimize collateral flow to the spinal cord. Figueroa and associates reported on 290 patients treated by FB-EVAR in the United States Aortic Research Consortium.^10^ In that study, aneurysm sac enlargement was noticed in 15% of patients, and persistent type II endoleak at 1 year was significantly associated with sac enlargement.^10^ Giuffre et al^11^ reported significant remodeling of the aorta with FL embolization using FL plug. The technique of SMA or target vessel FL embolization may be complementary to these strategies by allowing improved seal zone and eliminating or reducing the risk of endoleak.

The IMPEDE-FX (Shape Memory Medical) embolization plug is a porous, biocompatible, and noninflammatory polymeric scaffold that has the ability to self-expand from its crimped state into its memorized shape by exposure to an aqueous environment and body temperature, creating a high surface area stasis and promoting stable thrombus formation followed by collagen deposition. The polymer does not promote a chronic inflammatory reaction and has low radial force, which is optimal when used alongside self-expanding stents to promote remodeling of the target vessel. Another potential advantage is that the polymer does not add artifact that precludes visualization of surveillance imaging studies.^12^

## Conclusions

In patients with chronic PD-TAAAs undergoing staged FB-EVAR repair, adjunctive FL embolization of the SMA may allow optimization of target vessel sealing zone in patients who are considered unsuitable for FB-EVAR. This preliminary experience on three patients included successful sealing without type IC endoleak or target vessel compromise. Larger clinical experience and longer follow-up are needed to determine the durability of this adjunctive maneuver.

## Author contributions

Conception and design: ZF, HO-B, YH, SM, NS, EG, TM, GO

Analysis and interpretation: ZF

Data collection: ZF, HO-B

Writing the article: ZF, GO

Critical revision of the article: ZF, HO-B, YH, SM, NS, EG, TM, GO

Final approval of the article: ZF, HO-B, YH, SM, NS, EG, TM, GO

Statistical analysis: ZF

Obtained funding: Not applicable

Overall responsibility: GO

## Funding

This study was sponsored by Grant from the 10.13039/100019408Marcus Foundation and The Baylor Center for Aortic Surgery Marcus VITAL Laboratory.

## Disclosures

G.S.O. serves as consultant and/or scientific advisory board member for WL Gore, Cook Medical Inc., Centerline Biomedical, GE Healthcare, ViTAA Medical Solutions, Archo Medical, Shape Memory Medical, TripleMed B.V., and Artivion; All consulting fees, proceeds, and research grants are paid to Baylor College of Medicine, and the author receives no personal income.
